# Rapidly Progressing Renal Cell Carcinoma With Unexpected Inferior Vena Cava Tumor Thrombus

**DOI:** 10.1002/iju5.70014

**Published:** 2025-03-12

**Authors:** Sohei Iwagami, Masaya Nishihata, Shimpei Yamashita, Isao Hara, Fumiyoshi Kojima, Yasuo Kohjimoto

**Affiliations:** ^1^ Department of Urology Kishiwada Tokushukai Hospital Kishiwada Japan; ^2^ Department of Urology Wakayama Medical University Wakayama Japan; ^3^ Department of Human Pathology Wakayama Medical University Wakayama Japan

**Keywords:** rapid progression, renal cell carcinoma, surgery, tumor thrombus

## Abstract

**Introduction:**

We report here a case of RCC without preoperative tumor thrombus that had progressed to RCC with IVC tumor thrombus.

**Case Presentation:**

A 75‐year‐old man was referred to our hospital. Contrast‐enhanced CT showed a 50‐mm mass in the right kidney with an indistinct border, contrast in the early phase and washout in the late phase. RARN was attempted for RCC. Intraoperatively, a tumor thrombus was unexpectedly observed. We converted to open surgery, and the right kidney and tumor thrombus were removed. Postoperative lymphorrhea was observed, but the patient recovered without any problems. Lung and bone metastases subsequently appeared, and the patient died 2 months later.

**Conclusions:**

Surgeons should keep in mind that a tumor thrombus can grow rapidly before performing surgery for renal cell carcinoma.

**Abbreviations & Acronyms**
CT: computed tomography
IVC: inferior vena cava
RARN: robotic‐assisted Radical nephrectomy
RCC: renal cell carcinoma

**Summary**

- Venous tumor thrombus in renal cell carcinoma is occasionally encountered and can grow rapidly.
- Imaging evaluation at the appropriate time can help determine a safer surgical approach for the patient.

## 1 Introduction

RCC accounts for approximately 3% of all cancers and is characterized by the invasion of contiguous venous structures, particularly the renal veins and IVC [1]. IVC tumor thrombus is present in 4%–10% of RCC at the time of diagnosis [2, 3].

European guidelines recommend surgery as the gold standard for RCC with IVC tumor thrombus without metastases [1]. In addition to radical nephrectomy, tumor thrombus removal must be performed, and the method and difficulty of surgery are defined by the level of tumor thrombus [2, 4]. Therefore, it is essential to accurately determine the level of tumor thrombus preoperatively.

Recently, cases of RCC with IVC tumor thrombus that was preoperatively Level 2 rapidly increased to Level 3 within 6 weeks were reported [5, 6]. Clinical surgeons occasionally encounter RCC with rapidly progressing IVC tumor thrombus. We report here a case of RCC without preoperative tumor thrombus that had progressed to RCC with Level 2 IVC tumor thrombus within 7 weeks.

## 2 Case Presentation

**FIGURE 1:**
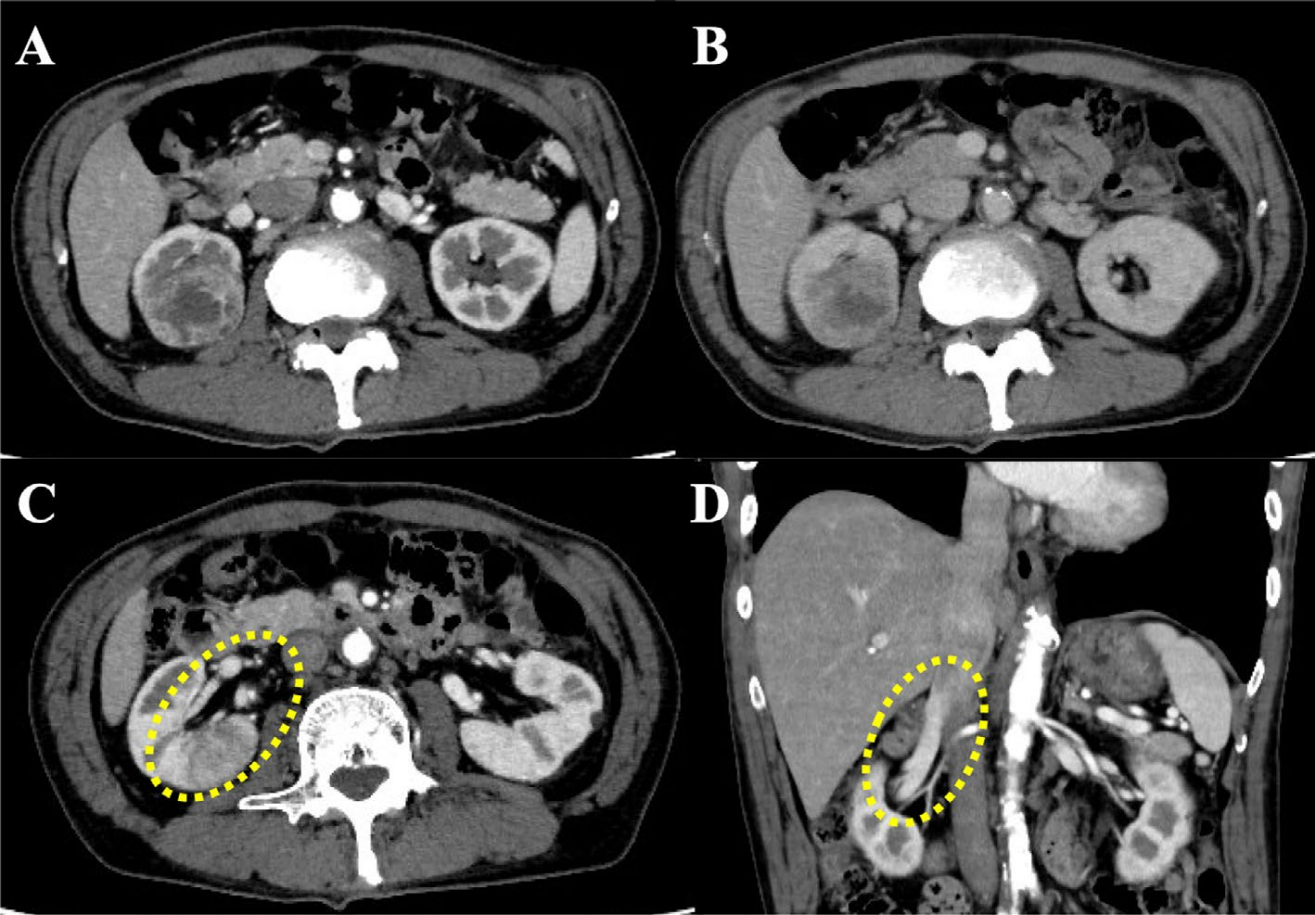
Computed tomography (CT) findings. (A: arterial phase, B: equilibrium phase) CT showed a tumor in the upper pole of the right kidney. (C) There was no tumor thrombus in the outflow vein (yellow dotted circle). (D) There was no tumor thrombus in the renal vein (yellow dotted circle).

**FIGURE 2:**
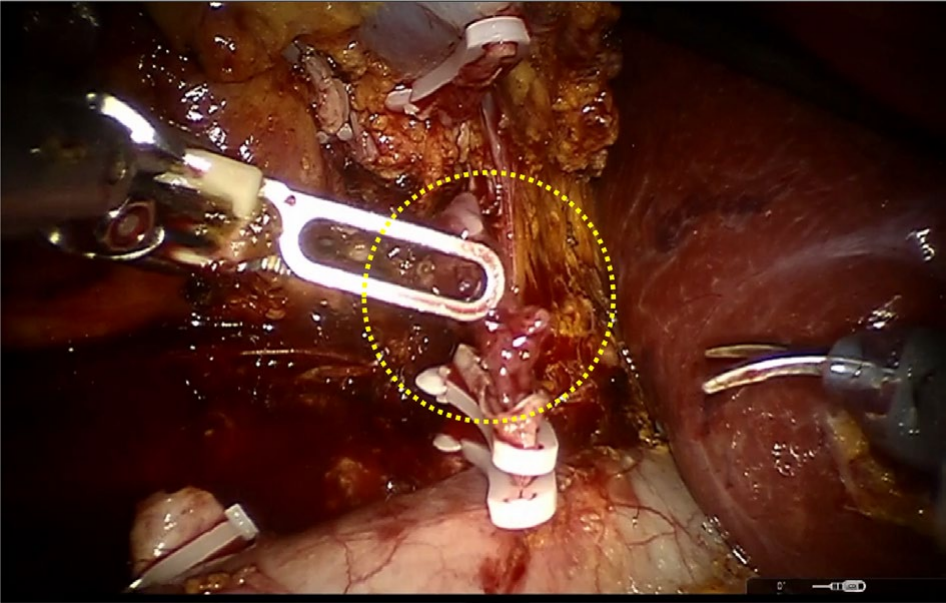
Intraoperative findings. When the renal vein was dissected, an intravenous tumor thrombus was observed (yellow dotted circle).

**FIGURE 3:**
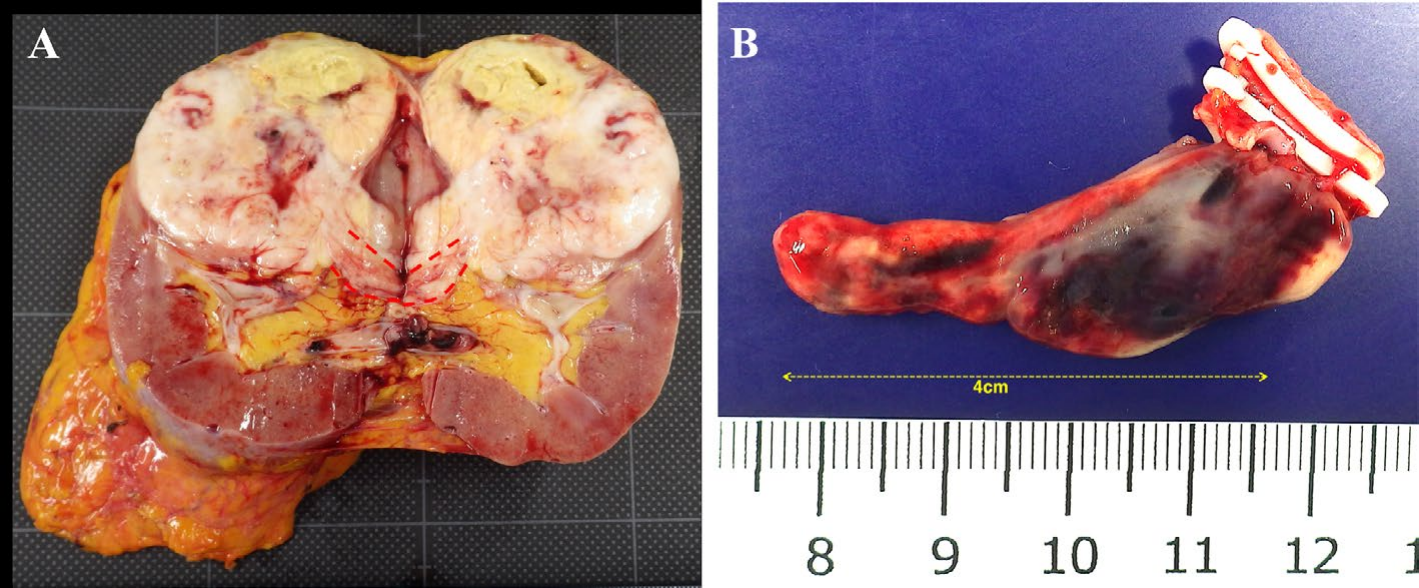
Resected specimen findings. (A) Red dotted line showed renal vein thrombus. (B) IVC tumor thrombus extending 4 cm cephalad from the renal vein.

A 75‐year‐old asymptomatic man presented to our hospital with incidental right renal cancer. The patient had a performance status of 0 and a medical history of hypertension. A contrast‐enhanced CT scan showed a 50‐mm tumor in the right kidney with an indistinct border, contrasted in the early phase and washed out in the late phase. There was no apparent tumor thrombus in the outflow and renal veins from the tumor and no apparent infiltration of the renal sinus (Figure 1). With the diagnosis of right renal cancer (cT1bN0M0), a robotic‐assisted nephrectomy (RARN) was performed 7 weeks after the CT scan. Intraoperatively, when the renal vein was dissected, a tumor thrombus was found in the lumen of the vessel (Figure 2). Ultrasonography was performed and revealed an RCC with Level 2 IVC tumor thrombus as high as the inferior border of the liver, but the level of the tip could not be confirmed. We converted from RARN to open surgery with the thoracic surgeon. After removing the right kidney, the IVC was incised, and the tumor thrombus was identified. No adhesion between the tumor thrombus and IVC was observed, and the tumor thrombus was removed with the renal vein. The IVC clamping time was 2 min. The operative time was 280 min, and the blood loss was 150 mL. The tumor diameter was 70 mm, and the IVC tumor thrombus grew 40 mm cephalad from the renal vein in the excised specimen (Figure 3).

**FIGURE 4:**
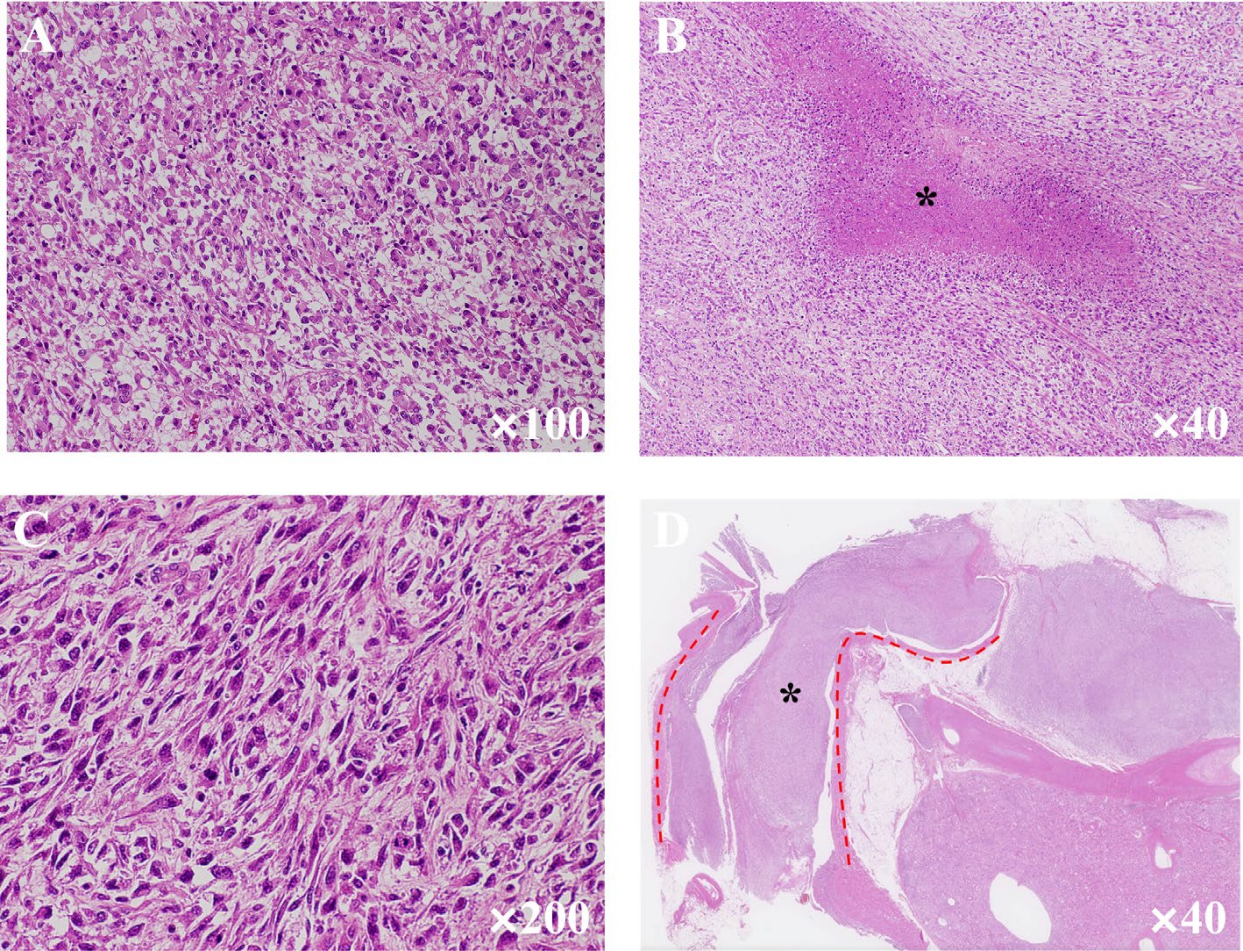
Pathological findings. (A) The tumor is composed of pleomorphic eosinophilic cells. The tumor is a high grade, poorly differentiated carcinoma, unclassifiable. (B) The tumor has foci of necrosis (asterisk). (C) Sarcomatoid change. The spindle cells are arranged haphazardly. (D) Tumor thrombus (asterisk) in the renal vein (red dotted line).

Histopathological examination revealed that the resected tumor was a high‐grade, poorly differentiated, unclassified RCC with necrosis and sarcomatoid changes (Figure 4). Pathologic T stage was pT3b with negative resection margins. We recommended adjuvant immune checkpoint inhibitor therapy postoperatively, but the patient declined treatment. Thereafter, lung and bone metastases rapidly appeared, and cancerous pleurisy developed. Eventually, respiratory failure developed, and the patient died 2 months later.

## 3 Discussion

In this case, a tumor thrombus that was not identified preoperatively was unexpectedly found intraoperatively. We decided to convert from RARN to open surgery in case open thoracotomy or the liver needed to be mobilized. On reflection, we overlooked the tumor thrombus in the renal vein during the vascular clip. Had we noticed it, we believe we should have performed an intraoperative ultrasound. The presence of tumor thrombus in the renal vein and IVC is highly relevant to surgery in renal cell carcinoma. This is because differences in surgical practices, invasiveness, and complications are likely to occur. To our knowledge, this is the first report of RCC with IVC tumor thrombus during RARN for RCC with no preoperative tumor thrombus identified in Japan.

Katsu et al. reported a case of IVC tumor thrombus that was Level 2 in the MAYO classification on preoperative MRI and progressed to Level 3 in 6 weeks [5]. Froehner et al. reported a case of Levels 1–2 IVC tumor thrombus that progressed to Level 3 within 1 month [6]. They considered Fuhrman grade 4 as the risk of the rapid progression of a tumor thrombus [5]. While the two reports described clear cell RCC, the present case described an unclassified RCC with necrosis and sarcomatoid changes. Recently, the natural growth rate of venous tumor thrombus was reported to average 0.3 mm/d, and rhabdoid/sarcomatoid differentiation showed faster growth (0.63 mm/d, *p* = 0.038) [7]. It is suggested that tumor thrombus grows more rapidly in RCC with sarcomatoid changes, as in the present case, even in the absence of a preoperative tumor thrombus.

Most RCC with sarcomatoid changes and unclassified RCC with high‐grade histologic features are associated with poor prognosis and may progress rapidly [8, 9]. These pathological features may be related to the rapid growth of a tumor thrombus. Unfortunately, due to the paucity of data, no obvious test can screen for these preoperatively, and radiological findings on imaging studies are not specific. In the present case, the contrast pattern was similar to that of clear cell RCC, but the contrast effect was low and borderline indistinct and the tumor might not be a typical renal cell carcinoma. However, there was no clear evidence of sarcomatoid changes or unclassifiable type. Further accumulation of data is expected in the future.

Appropriately timed imaging is the most important thing in evaluating the progression of venous tumor thrombus in RCC. In the present case, the preoperative CT may have missed the microvenous invasion between slices, and a repeat preoperative examination would have allowed for optimal treatment selection. Contrast‐enhanced CT with thin slices and multiple examinations would be necessary to avoid missing it. According to previous reports, preoperative imaging evaluation within 14–30 days was recommended in cases of venous tumor thrombus [10, 11]. It should be recognized that even in the absence of a preoperative tumor thrombus, Relatively large RCCs with atypical imaging findings may have microvenous invasion and may grow rapidly. Considering the additional financial burden and hassle, Additional imaging rechecks or ultrasounds may be useful if the waiting period before surgery exceeds 1 month.

Occasionally, in RCC, a tumor thrombus may grow quickly, even in the absence of preoperative evidence of venous tumor thrombus. Properly timed imaging evaluation is essential because inaccurate preoperative evaluation can harm the patient.

## Consent

Written informed consent was obtained from the patient for the publication of this case.

## Conflicts of Interest

The authors declare no conflicts of interest.
